# Effect of intra-alveolar placement of 0.2% chlorhexidine bioadhesive gel on the incidence of alveolar osteitis following the extraction of mandibular third molars. A double-blind randomized clinical trial

**DOI:** 10.4317/medoral.20009

**Published:** 2014-12-05

**Authors:** Josep Rubio-Palau, Jordi Garcia-Linares, Juan-Antonio Hueto-Madrid, Javier González-Lagunas, Guillermo Raspall-Martin, Javier Mareque-Bueno

**Affiliations:** 1 MD, DDS, PhD. Hospital Clínic de Barcelona Barcelona, Spain; 2 MD, DDS PhD. Hospital Germans Trias i Pujol Badalona, Spain; 3 MD, Hospital Universitari Vall d’Hebron Barcelona, Spain; 4 MD, DDS, PhD. Hospital Quirón Barcelona, Spain; 5 MD, DDS, PhD. Universidad CEU San Pablo de Madrid Madrid, Spain; 6 MD, DDS, PhD. Universitat Internacional de Catalunya Barcelona, Spain

## Abstract

Alveolar osteitis (AO) is a common complication after third molar surgery. One of the most studied agents in its prevention is chlorhexidine (CHX), which has proved to be effective. Objectives: The aim of this randomized double-blind clinical trial was to evaluate the efficacy of 0.2% bioadhesive chlorhexidine gel placed intra-alveolar in the prevention of AO after the extraction of mandibular third molars and to analyze the impact of risk factors such as smoking and oral contraceptives in the development of AO. Study Design: The study was a randomized, double-blind, clinical trial performed in the Ambulatory Surgery Unit of Hospital Vall d’Hebron and was approved by the Ethics Committee. A total of 160 patients randomly received 0.2% bioadhesive gel (80 patients) or bioadhesive placebo (80 patients). Results: 0.2% bioadhesive chlorhexidine gel applied in the alveolus after third molar extraction reduced the incidence of dry socket by 22% compared to placebo with differences that were not statistically significant. Smoking and the use of oral contraceptives were not related to higher incidence of dry socket. Female patients and the difficulty of the surgery were associated with a higher incidence of AO with statistically significant differences. 0.2% bioadhesive chlorhexidine gel did not produce any of the side effects related to chlorhexidine rinses. Conclusions: A 22% reduction of the incidence of alveolar osteitis with the application of 0.2% bioadhesive chlorhexidine gel compared to placebo with differences that were not statistically significant was found in this clinical trial. The lack of adverse reactions and complications related to chlorhexidine gel supports its clinical use specially in simple extractions and adds some advantages compared to the rinses in terms of duration of the treatment and reduction of staining and taste disturbance.

** Key words:**Alveolar osteitis, dry socket, chlorhexidine bioadhesive gel, mandibular third molar surgery.

## Introduction

Alveolar osteitis (AO) is a common complication after the extraction of teeth that was first defined in 1896 by Crawford. There are different terms to refer to this condition such as dry socket, localized osteitis, postoperative alveolitis, alveolalgia, alveolitis sicca dolorosa, septic socket, necrotic socket, localized osteomyelitis or fibrinolytic alveolitis. (1) The average incidence for all dental extractions ranges from 0.49% to 68.1% and in third molar surgery ranges from 1% to 30% (2,3).

There are different subjective clinical symptoms and Blum standardized the definition for AO: postoperative pain in an around the extraction site, which increases in severity at any time between 1 and 3 days after the extraction accompanied by a partially or totally disintegrated blood clot within the alveolar socket with or without halitosis (1). The cause of AO has not been established but Birn investigated the role of a locally increased fibrinolytic activity in its pathogenesis and it is believed to have a multi factorial origin with several agents like oral microorganisms, difficulty and trauma during extractions, roots or bone fragments remaining in the wound, excessive curettage of the alveolus, the use of anesthesia with vasoconstrictor, oral contraceptives, or smoking (1).

Systemic antibiotics have proved to be effective in reducing the frequency of AO and wound infection after third molar surgery, (4) but the increasing rate of adverse reaction to antibiotics (around 6-7% of the patients) (5) and bacterial resistance has lead to investigate new treatments. Several pharmacological interventions have been studied in the prevention of AO such as antibacterial agents, antifibrinolytic agents, antiseptic agents, obtundent dressings, steroid-anti-inflammatory agents, clot-support agents and recently plasma rich in growth factors (1,6). The antiseptic rinse most studied is chlorhexidine which has reported reductions in alveolar osteitis rates from 24.5% up to 80.2%. (1,7-11). Recently some investigations have studied the effect of 0.2% bioadhesive chlorhexidine gel with reductions of 60-70% in the incidence of AO (12-17).

The aim of this study is to evaluate the efficacy of 0.2% bioadhesive chlorhexidine gel in the prevention of AO after the extraction of mandibular third molars and to analyze the impact of risk factors such as smoking and oral contraceptives in the development of AO.

## Material and Methods

The study was a randomized, double-blind, placebo-controlled, single-center, parallel-group clinical trial conducted at the Minor Outpatient Surgery Unit of our Hospital from april 2008 until november 2010.

The sample size was calculated previously in collaboration with the Statistics Department with the program Epi-info, with 80 patients treated with chlorhexidine gel and 80 with placebo, with a significance level of 5% and a statistical power of 80% to detect as significant a difference corresponding to an incidence of 11% in the chlorhexidine group and 30% in the placebo group.

The study was approved by the Ethics Committee of the Hospital in December 2007. All the patients received prior to the intervention a document that described the study and signed an Informed Consent following the Principles of the Helsinki Declaration.

Chlorhexidine (0.2% Bioadhesive Chlorhexidine gel) and placebo gels were provided by Laboratorios Lacer (C/ Sardenya 350, Barcelona, Spain) in white single dose tubes of 10ml. Randomization of patients was performed by the Department of Statistics by a random list grouping the total of 160 patients in groups of 4 so that the distribution of the two groups (chlorhexidine and placebo) were homogeneous throughout the sample.

Clinical history of the patient was done noting the sex, age, phone number, use or not of oral contraceptives, smoking (if so also the amount), medical history, regular medication and reason for extraction. Ortopantomography was used in order to classify the difficulty of the extraction according to Koerner’s index, a scale that classifies the difficulty of the extractions according to the position of the third molar in the Winter classification (mesio-angular, disto-angular, horizontal or vertical) and the relationship between the impacted lower wisdom tooth to the ramus of the mandible and the 2nd molar in the Pell and Gregory classification and were included the extractions with a difficulty index between 4 and 7. Exclusion criteria were unwillingness to participate, patients with pericoronitis, active infection or antibiotic treatment at the time of surgery or in the last two weeks, to avoid false results due to the presence of active infection, patients with significant systemic disease, pregnancy, immunocompromised patients, AIDS or with associated bone pathology. Patients that were taking oral contraceptives or smokers were not excluded.

The operation was performed under local anesthesia with inferior alveolar and lingual nerves block and local infiltration. The anesthetic used was 40 mg articaine with epinephrine hydrochloride 0.05% (Ultracain®, Laboratorios Normon, Madrid. Spain). The extraction was done with elevators when it was simple or by removing the surrounding bone and sectioning the tooth and the roots when it was necessary. After rinsing the socket with saline and gentle curettage, a single dose of bioadhesive gel (0.2% chlorhexidine or placebo according to the random list and neither the surgeon nor the patient knew the substance) was introduced and posteriorly suture with vicryl 4/0. Finally a compressive gauze was introduced during 30-45 minutes and cold was applied locally. A document with the postoperative instructions and treatment was given (diclofenac 50 mg every 8 hours alternated with metamizol 575 mg every 8 hours and omeprazole 20 mg per day without postoperative administration of bioadhesive gel). Epidemiologic and intraoperative data such as age, gender, smoking, oral contraceptives, difficulty of the extraction, side of the extraction, cause of the extraction, type of operation or surgical time were collected.

At the second or third postoperative day a first control was done in the office and a second control was done after 7-8 days in order to diagnose an AO according to the following criteria: uncontrollable pain with the analgesia prescribed between 1 and 3 days after the extraction with one or more of the following: partial or total disintegration of the clot, detritus, empty socket/exposed alveolar bone +/- halitosis. When a dry socket was diagnosed the following measures were implemented: Realization of a microbiological culture, careful irrigation with chlorhexidine digluconate mouthwash 0.12%, application of bioadhesive chlorhexidine gel 0.2%, suture removal (if necessary in some cases due to difficulty of hygiene producing a valve mechanism in the gum allowing entry of detritus in the socket but not exit), variation of the medication with the introduction of an antibiotic: amoxicillin-clavulanate or clindamycin if allergic to penicillin. Possible complications and treatment intolerance were noted. A Chi-Square test was used when the expected frequencies were greater than 5, otherwise a Fisher test was used. When the dependent variable was qualitative ordinal the statistic test used was Kendall’s Tau-b. It was considered as statistically significant differences for 5% significance level .

## Results

160 patients were studied, 80 of them received 0.2% bioadhesive chlorhexidine gel and the other 80 a placebo gel ( Table 1). Of the 160 patients, 53.8% (86) were women and the mean age was 25.04 years. 26 out of 86 women were taking oral contraceptives and 67 patients were smokers (41.80%). The difficulty of the tooth extraction according to Koerner’s scale was 4: 49 patients (30.60%), 5: 49 patients (30.60%), 6: 42 patients (26.25%) and 7: 20 patients (12.50%) and the third molar extraction was simple (with elevators) in 61 patients (38.12%) and surgical (bone removal +/- root sectioning) in 99 patients (61.87%). 118 patients tolerated the treatment (73.75%) and 42 did not (26.25%) with gastrointestinal discomfort in 30 patients, dizziness in 10 patients and skin rash in 2 of them. The complications of the procedure were temporal paresthesia of the inferior alveolar nerve in 4 patients (2.5%), 3 phlegmons (1.87%), 1 socket bleeding (0.62%) and 1 TMJ pain (0.62%). 14 patients with AO received chlorhexidine gel (17.50%) and 18 patients with AO received placebo (22.50%) (X2 Pearson = 0,625; *p* = 0,554), this represented a 22.22% reduction in the occurrence of AO in the chlorhexidine group but statistical differences were not significant ( Table 2). No statistical differences were detected in the incidence of AO in patients that were taking oral contraceptives or smokers. A significant association in the degree of difficulty was observed. If the difficulty of the extraction was 4, a 10% of alveolar osteitis was found, whereas if the difficulty was 5 or greater alveolar osteitis was observed in percentages above 20%.

**Table 1:**
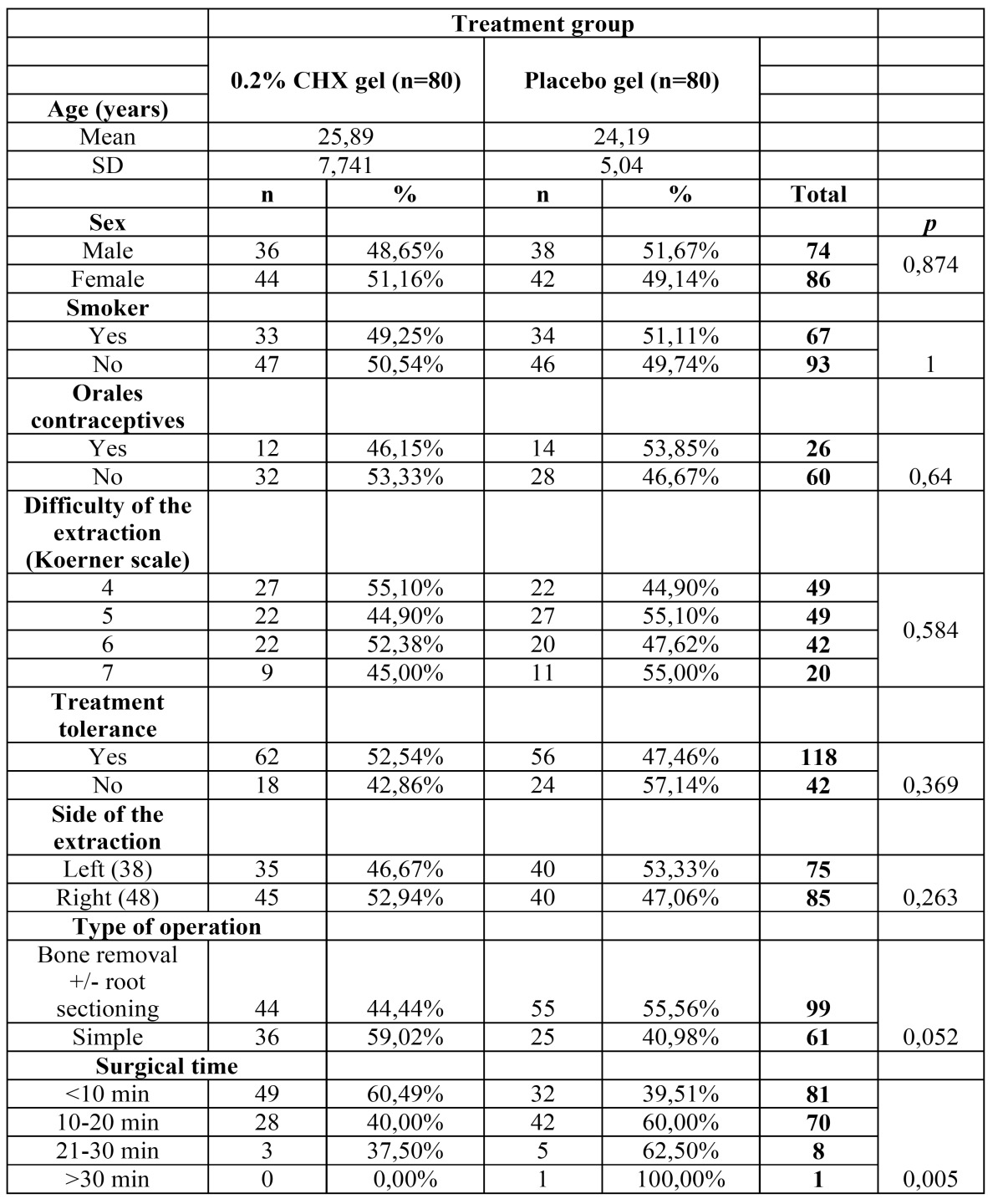
Description of patients in each group.

**Table 2:**
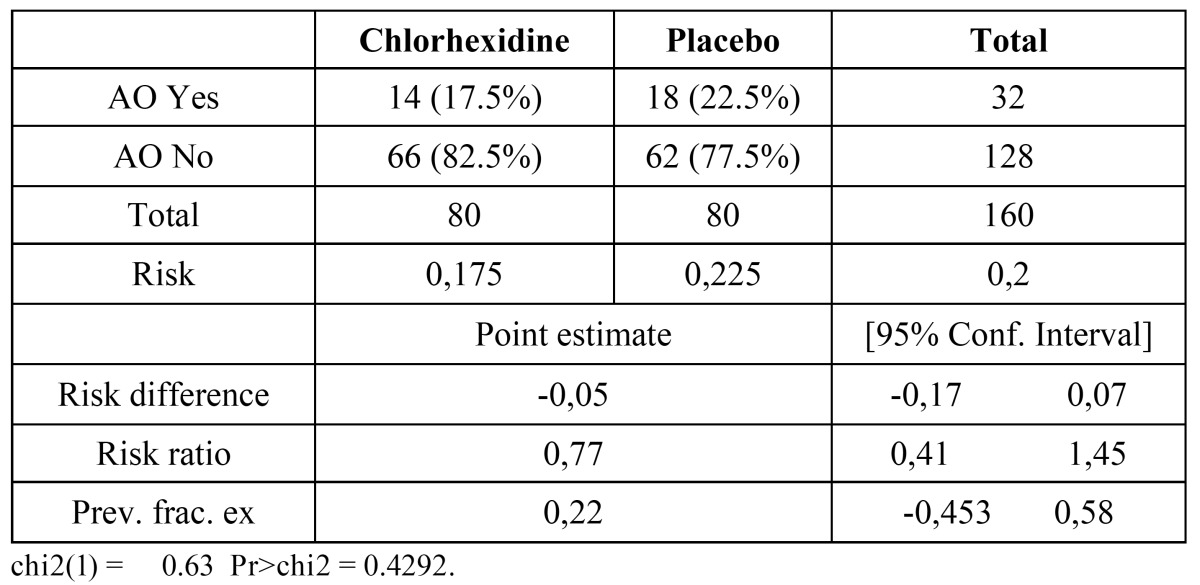
Risk results of AO in the 0.2% bioadhesive chlorhexidine and the placebo groups.

## Discussion

The results of this study revealed that the intra-alveolar placement of 0.2% bioadhesive chlorhexidine gel might reduce the frequency of alveolar osteitis in 22.22% compared to the control group. There are few papers that have studied the effectiveness of the biadhesive chlorhexidine gel in the prevention of alveolar osteitis. Torres-Lagares et al found a 42.65% reduction in a pilot study with 30 patients (16) a 63.33% reduction in a sample of 103 patients (12), and posteriorly a 57.15% reduction in 38 patients with bleeding disorders (15), Hita-Iglesias *et al* (13) observed a 70% reduction in a study that compared effectiveness of chlorhexidine gel versus chlorhexidine rinse in 73 patients, Haraji found a 65.4% reduction in a split-mouth clinical trial with 80 patients (14). Rodríguez-Pérez *et al* (17) studied the effectiveness of chlorhexidine gel at 0.2% and 1% and observed that there were no significant differences in AO after surgical extraction of mandibular third molars.

The effectiveness of the chlorhexidine mouthwash during the surgery and after one week has been demonstrated in several studies. Hermesch *et al* found a 44.2% reduction (3), Larsen a 60.3% reduction (8), Tjernberg a 80.2% reduction (9), Ragno and Szkutnik a 51.8% reduction (11), Bonine a 56% reduction (10) and Metin a 42% reduction (18). In a meta-analytic review by Caso (2) it was concluded that rinsing with chlorhexidine on the day of the surgery and several days after may reduce the incidence of AO. In a paper based on a Cochrane Review (19) concluded that there is some evidence that rinsing with chlorhexidine (0.12% and 0.2%) or placing chlorhexidine gel (0.2%) in the sockets of extracted teeth, provides a benefit in preventing dry socket. However, a recent systematic review by Yengopal (20) could not identify sufficient evidence supporting the use of chlorhexidine for the prevention of AO, and posteriorly Richards (21) reviewed its effectiveness and concluded that there is insufficient evidence on which to recommend the use of chlorhexidine to prevent alveolar osteitis.

The difficulty of the extraction (according to Koerner’s scale) was higher than other studies (12,13) and 61.8% of the extractions were surgical. Wet her if the extraction was simple or surgical and the duration of the operation (two important factors in the pathogenesis of AO) are not described in other studies and this could be one reason of the lower reduction of the incidence of AO of this clinical trial. A higher incidence of alveolar osteitis was found in the surgical extractions (23.23%) than in simple extractions (14.75%) *p*=0.226, as well as operations longer than 10 minutes had higher incidence of alveolar osteitis (21.43-25%) than shorter than 10 minutes (17.28%) *p*=0.284 but statistically differences were not significant.

Risk factors associated to AO such as smoking (41.9% of the patients) or oral contraceptives (30.2% of women) did not influence in this study. These results are similar to other recent studies with chlorhexidine gel by Torres-Lagares (12,16) and Hita-Iglesias (13) that did not find statistically difference regarding these risk factors. Hermesch (3) observed that smoking was not related to a statistically significant increase of AO but there was a 47% increase in the incidence of AO in women taking oral contraceptives.

None of the patients of this study complained about teeth or mucosa staining, mucosa erosion, taste disturbance or parotitis which are related to chlorhexidine rinses. (18,22) This local effect of the gel without alteration to the rest of the oral cavity and the longer effect due to it is bioadhesive are some advantages of the gel in comparison to the mouthwash.

The sample size of the study was appropriate in order to assess the effects of intra-alveolar placement of chlorhexidine gel on the incidence of AO and is the longest published with chlorhexidine gel. Haraji (14) also studied a total of 160 mandibular third molars extractions but in 80 patients in a split-mouth clinical trial. Torres-Lagares series studied 30 (16), 103 (12) and 38 (15) patients, Hita-Iglesias (13) 73 patients and Rodríguez-Pérez (17) 88 patients.

A 26.25% of the patients did not tolerate the postoperative treatment due to gastrointestinal discomfort associated to metamizol in the majority of them which was solved by changing the analgesic to paracetamol 1g. 5.6% of the patients suffered complications that could be treated without any sequela, which was similar to Hermesch (3).

The study was balanced in terms of sex, age, smoking, oral contraceptives, difficulty of the extraction and side of the extraction. The almost equal number between male and female patients was taken in consideration in Haraji (14) and Rodríguez-Pérez (17) studies, but not in Torres-Lagares (12,16) and Hita-Iglesias (13) studies which present a higher proportion of females. We have found a higher incidence of AO in women (29.07%) than in men (9.46%) *p*=0.003 which could be interpreted as the female sex might be a risk factor of AO (1,23). This is a very important issue that has not been described in other studies which probably with a balanced proportion of males and females would have lower rates of AO. (12,13,16) The mean age of the patients was similar to other studies (12,13,17) and the taking of oral contraceptives (30.23%) is higher than other similar studies that have an incidence around 14% (12,13). This was considered a risk factor of AO (1) and some studies with chlorhexidine rinse found an increase in AO in females using oral contraceptives (3,10) but other studies with chlorhexidine gel did not find any difference (12,13). Smoking did not increase the incidence of AO in this study. Hita-Iglesias (13) and Hermesch (3) did not find an increase of AO in smokers, but the majority of the studies observed a higher incidence of AO in smokers (1,10,12,18).

There are many studies and reviews that support that chlorhexidine rinses and gel reduce the incidence of AO after the extraction of mandibular third molars. A 22% reduction of the incidence of alveolar osteitis with the aplication of 0.2% bioadhesive chlorhexidine gel compared to placebo with differences that were not statistically significant was found in this clinical trial. The lack of adverse reactions and complications related to chlorhexidine gel supports its clinical use in simple extractions and adds some advantages compared to the rinses in terms of duration of the treatment and reduction of staining and taste disturbance.
